# Meta-Analysis of the Long Term Success Rate of Different Interventions in Benign Biliary Strictures

**DOI:** 10.1371/journal.pone.0169618

**Published:** 2017-01-11

**Authors:** Orsolya Huszár, Bálint Kokas, Péter Mátrai, Péter Hegyi, Erika Pétervári, Áron Vincze, Gabriella Pár, Patrícia Sarlós, Judit Bajor, József Czimmer, Dóra Mosztbacher, Katalin Márta, Csaba Zsiborás, Péter Varjú, Ákos Szücs

**Affiliations:** 1 Semmelweis University, 1^st^ Department of Surgery, Budapest, Hungary; 2 Institute of Bioanalysis, University of Pécs, Medical School, Pécs, Hungary; 3 Institute for Translational Medicine, University of Pécs, Pécs, Hungary; 4 Division of Translational Medicine, First Department of Medicine, University of Pécs, Pécs, Hungary; 5 Hungarian Academy of Sciences - University of Szeged, Momentum Gastroenterology Multidisciplinary Research Group, Szeged, Hungary; 6 Division of Gastroenterology, First Department of Medicine, University of Pécs, Pécs, Hungary; 7 1st Department of Paediatrics, Semmelweis University, Budapest, Hungary; Texas A&M University, UNITED STATES

## Abstract

**Background:**

Benign biliary stricture is a rare condition and the majority of the cases are caused by operative trauma or chronic inflammation based on various etiology. Although the initial results of endoscopic, percutaneous and surgical treatment are impressive, no comparison about long term stricture resolution is available.

**Aims:**

The goal of this study was to compare the long term disease free survival in benign biliary strictures with various etiology after surgery, percutaneous transhepatic—and endoscopic treatment.

**Methods:**

PubMed, Embase, and Cochrane Library were searched by computer and manually for published studies. The investigators selected the publications according to the inclusion and exclusion criteria, processed the data and assessed the quality of the selected studies. Meta-analysis of data of 24 publications was performed to compare long term disease free survival of different treatment groups.

**Results:**

Compared the subgroups surgery resulted in the highest long term stricture resolution rate, followed by the percutaneous transhepatic treatment, the multiple plastic stent insertion and covered self-expanding metal stents (SEMS), however the difference was not significant. All compared methods are significantly superior to the single plastic stent placement. Long term stricture resolution rate irrespectively of any therapy is still not more than 84%.

**Conclusions:**

In summary, the use of single plastic stent is not recommended. Further randomized studies and innovative technical development are required for improving the treatment of benign biliary strictures.

## Introduction

Benign biliary stricture is a rare condition and majority of the cases are caused by operative trauma, mainly after open, or laparoscopic cholecystectomy [1] [2]. The second most common cause is the fibrosis at the site of a surgical anastomosis [1]. Other conditions could also lead to benign bile duct obstruction like chronic pancreatitis, sclerosing cholangitis, cholelithiasis, impacted stones, sphincterotomy and infection of the biliary tract [3].

The symptoms vary in wide range from asymptomatic presence to complete obstruction with jaundice, pain, pruritus, biliary stones, cholangitis and biliary cirrhosis[3].

At the moment there are no ideal treatment for this disease, therefore come to a decision between the therapies is individual. The reocclusion rate is unacceptably high in all procedures, which is around 20% [2]. Real long term follow-up still does not exist making the comparison almost impossible.

The bottom line of surgical approach is to restore the bilio-digestive continuity. This could be reached by hepaticojejunostomy, choledochojejunostomy, or intrahepatic cholangiojejunostomy. The most preferable solution is hepaticojejunostomy in benign biliary strictures, however most of these patients are poor candidates for surgery due to malnutrition, cirrhosis, and portal hypertension [2, 4, 5].

The minimal invasive therapies mean repeatable interventions, which raise the amount of complications, such as cholangitis, bleeding or perforation. The type of the endoprothesis and the etiology of the stricture can also determine the result of the intervention. The most peripheral treatment of benign biliary stricture is balloon dilatation by endoscopic or percutaneous manner. Percutaneous access could worsen life quality thank to the drainage for a shorter period. Single plastic stents used for bridging biliary obstruction are easy to apply and inexpensive, but have insufficient diameter and therefore short stent patency, which is requiring replacement every 2–3 months. Therefore, a widely used and most preferable alternative is a multiple plastic stent insertion. It is very effective, but the stent patency is short and additional endoscopic procedures required. Covered self-expandable metal stent can achieve larger diameter, providing longer stent patency with less endoscopic interventions, but is reported to have 80% to 90% long-term success rate after stent removal, which is still unacceptably high in a benign disease with long life expectance [6]. Therewithal, self-expandable metal stent has several early and late complications, such as biliary infection, pancreatitis, bleeding, perforation, and particularly early stent migration[3] [5, 7].

Considering the benefits and disadvantages, the best choice currently for benign stricture is the usage of multiple plastic stents, however covered self-expendable metal stents show a promising future as well [8].

Running through the literature there are numerous studies about success rate and short term disease free survival of different therapeutic modalities, but no randomized multicentric studies exists where the long term results of different treatments could be compared. The aim of writing a meta-analysis on benign biliary disease, that this field in medicine is still unsolved and the question whether surgery, endoscopy, or transhepatic biliary intervention is the gold standard is still open.

## Materials and Methods

### Materials

All published journal articles, which were related to benign biliary stricture were searched in three main electronic databases, last search happened in 28.02.2016. It was not augmented with hand search. Computer research was done in databases of PubMed, Embase and Cochrane Library by three independent authors. The keywords were the following: benign biliary stricture, bile duct stricture, benign biliary obstruction, chronic pancreatitis, post-cholecystectomy, biliary stricture, biliary anastomosis, stent, surgery. (Database were narrowed by: benign biliary stricture AND stent AND surgery, chronic pancreatitis AND stent AND surgery, benign biliary obstruction AND surgery, benign biliary stricture AND stent, benign biliary stricture AND surgery, chronic pancreatitis AND stent, chronic pancreatitis AND surgery, post-cholecystectomy AND stent, post-cholecystectomy AND surgery, biliary stricture AND surgery, biliary stricture AND stent, biliary anastomosis AND surgery, biliary anastomosis AND stent, bile duct stricture AND stent, bile duct stricture AND surgery).

We excluded languages other than English. Based on the accelerated development of various endobiliary stents we decided to exclude publications about endoscopy before 2000. At the other therapeutic modalities there was no publication date restriction. Mesh words and free words were combined and the selection was performed manually.

### Methods

#### Inclusion criteria

Benign strictures were included only. The disease types were chronic pancreatitis, postoperative stricture and iatrogenic trauma. All three treatments were included: surgery, endoscopic and percutaneous intervention. We evaluated all types of stents and their use: single plastic stent, multiple plastic stent, metal stent and fully covered metal stent. The surgical methods were choledochoduodenostomy, choledochojejunostomy, hepatoduodenostomy and hepaticojejunostomy. Both retrospective and prospective studies were accepted. Only publications with at least one-year follow-up after the close of the intervention according every single patient (definitive removal of stent) were included.

#### Exclusion criteria

All studies were excluded where the follow-up after the close of the interventional period were shorter than one year. To accurately evaluate the studies just full texts were accepted. Follow-up time had to be clearly identified or calculated as disease free survival and follow up time. Poor quality, repeated reports and mistakable results were not accepted.

## Literature Screening, Quality Assessment, and Data Extraction

The literature was screened by the investigators independently, the quality of the studies was based on our inclusion and exclusion eligibility criteria. The data in all studies were then extracted before a cross-check of the results. The systematic review was conducted following the Preferred Reporting Items for Systematic Reviews and MetaAnalysis (PRISMA) guidelines[9].

**Fig 1:**
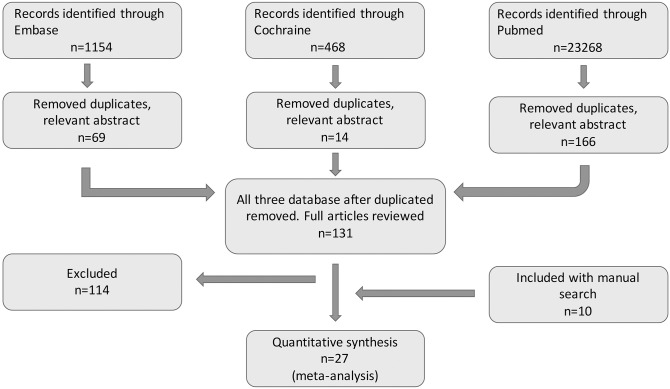
PRISMA diagram for the study.

According to the long term success rate numerous publications include data of patients into the final statistics who lost during the follow-up, other publications ignore that patients. Therefore, we reevaluated the long term success rates and patient numbers in order to get comparable results: we subtracted the number of the patients lost during the follow up from the number of patients followed and divided this number (Fig 1).

## Statistical Analysis

All meta-analysis were performed with random effect model using the Der Simonian and Laird method. Q-statistics and I^2^ indicator were calculated in each case to assess heterogeneity. If the Q test is significant (p<0.1) it implies that the effect sizes are more different from each other than it is expected due to random chance. In this case the diversity can be attributed to different clinical methods or the observed patients participating in the studies. I^2^ indicator shows the percentage of effect size variability that cannot be attributed to random chance but other factors mentioned above.

**Fig 2:**
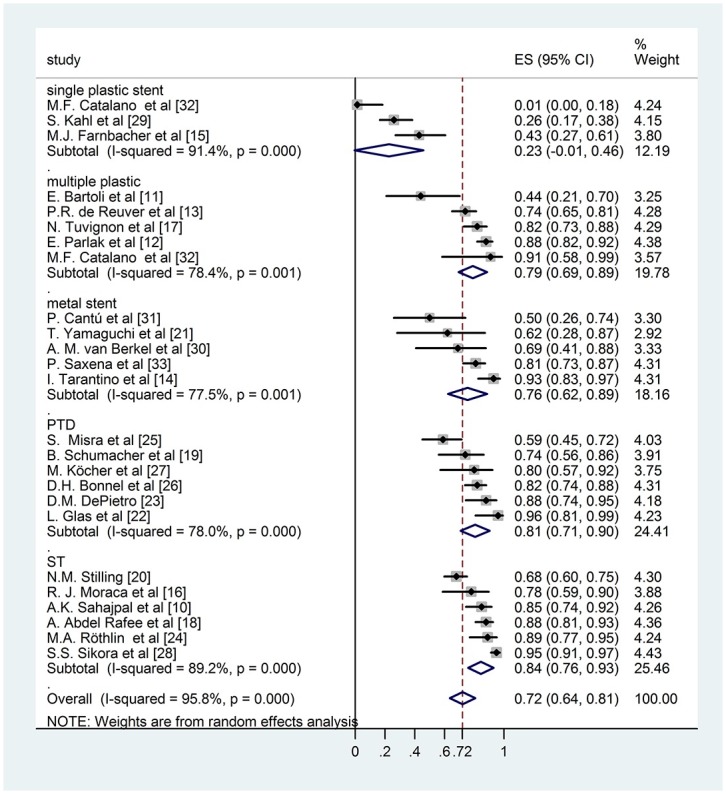
Forest plot comparing long term stricture resolution in different subgroups using modified rate with ordinary weighting.

**Fig 3:**
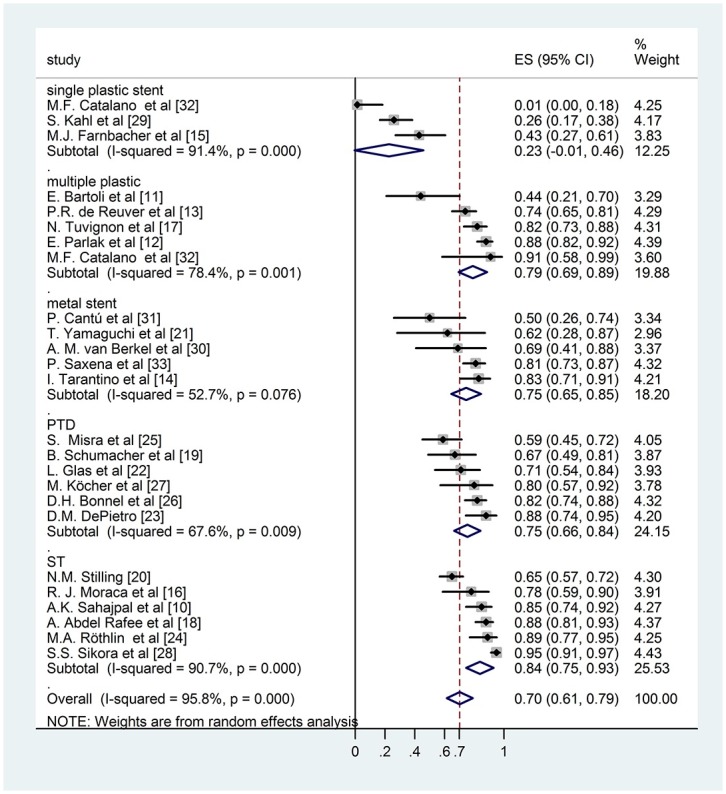
Forest plot comparing long term stricture resolution in different subgroups using originally published rate with ordinary weighting.

**Fig 4:**
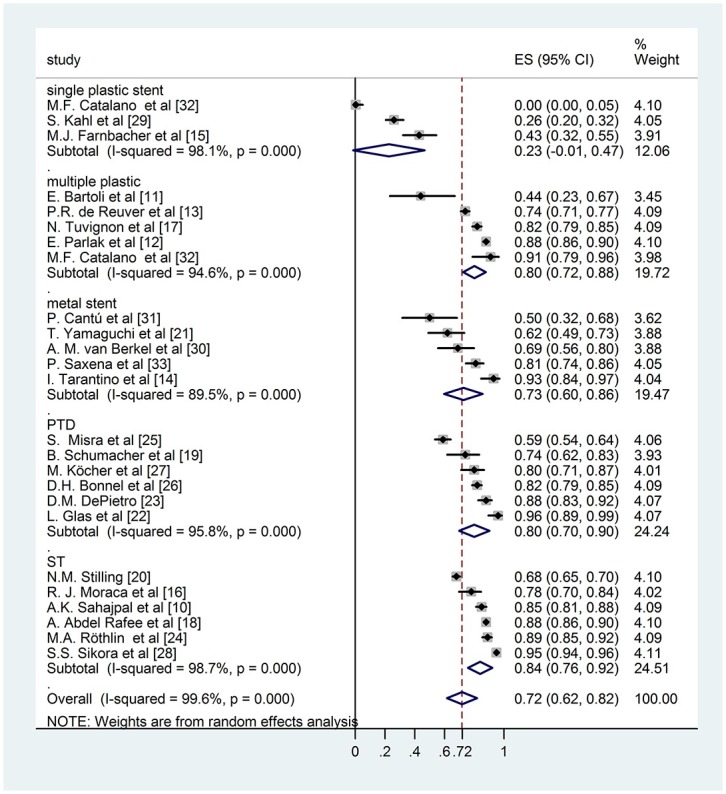
Forest plot comparing long term stricture resolution in different subgroups using modified rate with follow-up weighting.

**Fig 5:**
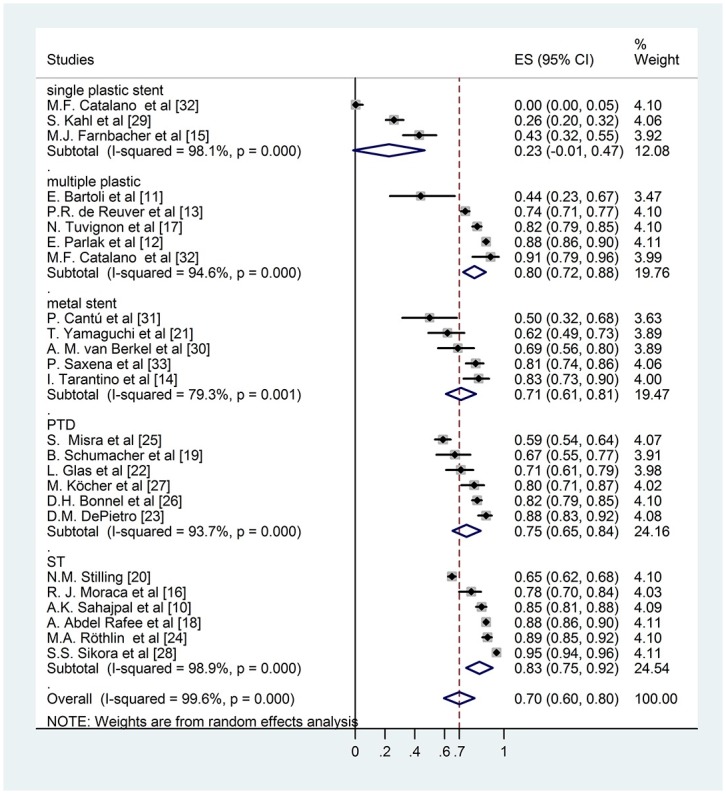
Forest plot comparing long term stricture resolution in different subgroups using originally published rate with follow-up weighting.

During the analysis we had to realize that the follow up times reported by the authors varied in a very wide range, even within the same study. The biggest challenge of this work was to handle this difficulty and investigate whether the results effect the final conclusion. We used an alternative weighting method as well along with the conventional random effect weighting procedure: we multiplied the sample sizes with the (mean or median) follow up years making it possible for the follow up time to contribute to the weights. Bigger sample size results in smaller standard error which yields a bigger weight to the specific study and it allows us to see how the result change if we take into account this information in the weights. Comparing the results of the conventional weighting (Figs 2 and 3) and the one altered by the follow up time (Figs 4 and 5), the conclusions are robust concerning this difference. The two weighting methods yields almost exactly the same estimates and therefore do not affect the conclusion of the analysis. To prove that the success rates show no association with the follow up times, we performed a meta regression. During this investigation we found that the result does not support the hypothesis that longer follow up time associates with bigger success rate (Coefficient of follow up time = 0.1 p = 0.18). We investigated the same question by the different techniques (subgroups) because even if there is no association concerning all of the studies there could be one in the different subgroups. However, the results show no association in either of the subgroups. In case of single plastic there were not enough studies to perform the meta regression (S2 Fig).

To compare the long term success rates of different treatments, we used subgroup analysis, p < 0.05 indicating significant difference

Finally, we tested the presence of publication bias using Egger’s test using p< 0.1 for detecting significant bias.

Comprehensive Meta-Analysis Software (Biostat Inc.) and Stata 11 SE (Stata Corp.) were used for the computations and graphs.

## Results

### Characteristics of the included studies

According to the inclusion and exclusion criteria a total of 24 articles were included in the present meta-analysis. One of the article contained two groups, which were calculated individually. 14 publications of them were retrospective cohort studies, 11 were prospective trials, one of them contained both retrospective and prospective results. No randomized controlled study was found.

The publications were divided into 3 main subgroups according to the therapeutic modality: 6 publications in surgery, 13 in endoscopic and 6 in percutaneous transhepatic treatment. Endoscopic interventions were further classified into 3 subgroups based on the stent material: 3 with single plastic stent, 5 with multiple plastic stent and 5 with covered metal stent insertion. (Table 1 [10–33])

**Table 1:** Patient Information of the Included Studies.

Manuscript	Single center (SC) / multi center (MC)	Year	Type of intervention *(endoscop—ET*, *percutaneous tranhepatic drainage—PTD*, *surgery ST)*	Stent type *(metal stent-MS*, *single plastic stent-SPS*, *multiple plastic stent-MPS)*	Number of patients	Not treated	Modified long term succes rate	Long term succes rate %	Lost during follow up	Number of patients in follow up	Long term follow up time (mean)	Long term follow up time (median)	SD	range
Payal Saxena et al	MC	2015	**ET**	MS	123	14	**81%**	81%	0	109		18,5 month		49-3month
Tarantino I et al	MC	2011	**ET**	MS	62	0	**93%**	83%	0	56		15,9 months	10 months	
A. M. van Berkel et al	SC	2003	**ET**	MS	13	0	**69%**	69%	0	13	50 months			6–86 months
P. Cantú et al	SC	2004	**ET**	MS	14	0	**50%**	50%	0	14		22months		12–33 months
Taketo Yamaguchi	MC	2006	**ET**	MS	8	0	**62%**	62%	3	8	88,8 months			78–99,6 months
N Tuvignon et al	MC	2011	**ET**	MPS	124	28	**82%**	82.30%	0	96		73,2 months		0,96–243,6 months
Erlan Parlak et al	SC	2014	**ET**	MPS	238	83	**88.40%**	89%	0	156		78 months		12–198 months
Philip R de Reuver et al	MC	2007	**ET**	MPS	110	0	**74%**	74%	0	110	91,2 months		44,4 months	
Eric Bartoli et al	MC	2005	**ET**	MPS	15	2	**44.40%**	44.40%	4	13	16 months			4–48 months
Catalano MF et al	SC	2004	**ET**	MPS	12	0	**91%**	91%	0	12	46,8 months			
S. Khal et al.	SC	2003	**ET**	SPS	69	8	**26%**	26.20%	0	61		40months		18–66 months
Farnbacher et al	MC	2000	**ET**	SPS	31	0	**43%**	43%	8	31	28months			
Catalano MF et al	SC	2004	**ET**	SPS	34	0	**0%**	0%	21	36	50,4 months			
Ludivine Glas et al	SC	2008	**PTD**		39	1	**96%**	71%	5	33	33,9 months			11,3–65,2 months
B. Schumacher et al	MC	2001	**PTD**		34	3	**74%**	67%	0	31		24,2 months	15,7 months	
Sanjay et misra	SC	2004	**PTD**		51	0	**58.80%**	58.80%	2	51		77 months	31 months	23–140 months
Daniel M. DePietro	SC	2015	**PTD**		71	18	**88%**	88%	0	42	56,4 months			0–144 months
Martin Köcher et al	SC	2007	**PTD**		21	1	**80%**	80%	0	20	62,4 months			16–132 months
Didier H.	SC	2012	**PTD**		111	1	**82%**	82%	37	110	59 months			0,5–278 months
Nicolaj M. Stilling	MC	2015	**ST**		139	0	**68%**	65%	7	139		114 months		0–182 months
Ahmed Abdalrafe et al	SC	2015	**ST**		120	no data	**88.30%**	88.30%	0	120		149 months		70–246 months
A.K. Sahajpal et. al.	MC	2010	**ST**		69	1	**85%**	85%	0	68		71,5 months		0–120 months
R. J. Moraca et al.	SC	2002	**ST**		27	0	**78%**	78%	0	27		54months		1–108 months
A.R. Markus et. al.	SC	1997	**ST**		51	1	**88.50%**	89%	15	50		91,2 month		2-13yrs
S.S. Sikora et al.	SC	2005	**ST**		245	5	**95%**	95%	0	225	90 months	81,6 months		25–187 month

### Publication bias analysis

The Egger's test showed no indication of publication bias (two sided p = 0,793) in long term success rates.

### Subgroup analysis of modified long term success rate

Six studies reported the long term disease free survival of surgical intervention. As shown in Fig 2, the weighted mean of the surgical group was (ES 0,84; 95% CI [0,76; 0,93]). Within the endoscopically treated group, the weighted long term success rate of 3 studies with single plastic stent insertion was (ES 0,23; 95% CI [-0,01; 0,46]), 5 studies with multiple plastic stent insertion was (ES 0,79; 95% CI [0,69; 0,89]) and 5 studies with covered metal stent was (ES 0,76; 95% CI [0,62; 0,89]). The pooled mean value of percutaneous transhepatic drainage proved to be (ES 0,81; 95% CI [0,71; 0,90]). These data do not differ significantly from data with follow-up weighting discussed previously (Fig 4).

Comparing the data of different groups by subgroup analysis shows no significant difference between surgical intervention, percutaneous transhepatic intervention and endoscopic multiple plastic stent or covered metal stent insertion (surgery vs. covered metal stent p = 0,19; surgery vs. multiple plastic stent p = 0,335; PTD—covered metal stent p = 0,342). However single plastic stent insertion indicates significantly worse long term disease free survival compared any other therapeutic modalities (covered metal stent—single plastic stent p = 0,001; multiple plastic stent—single plastic stent p< 0,001; PTD—single plastic stent p< 0,001; surgery—single plastic stent p< 0,001).

### Subgroup analysis of originally published long term success rate

Calculating with the previously presented subgroups we compared the long term success rate of different groups originally published in the publications but no difference was detected surgery—ES 0,84; 95% CI [0,75; 0,93], single plastic stent insertion—ES 0,23; 95% CI [-0,01; 0,46], multiple plastic stent insertion—ES 0,79; 95% CI [0,69; 0,89], covered metal stent insertion—ES 0,75; 95% CI [0,65; 0,85], percutaneous transhepatic intervention—(ES 0,75; 95% CI [0,66; 0,84]). (Fig 3). These data do not differ significantly from data with follow-up weighting discussed previously (Fig 5).

## Discussion

The benign biliary strictures represent a clinical diagnostic category which is extremely wide under many points of view. The clinically relevant approach is that all bile duct strictures in patients with obstructive jaundice should be considered malignant unless a benign etiology is definitively identifiable. However, the diagnostic arsenal has some uncertainty in store. ERCP or PTC with sampling is indispensable but it is limited by low sensitivity. The addition of FISH, Kras/p53 mutation analysis can give further important evidence that may help improve the diagnostic yield. EUS-FNA has been shown to be effective in diagnosing malignancy in patients with biliary strictures and should be considered as the initial endoscopic modality in all patients with suspected biliary strictures without obstructive jaundice. Use of intraductal ultrasound and cholangioscopy is limited due to the availability. Taking into consideration the above mentioned limitation of the diagnosis, publications selected for this meta-analysis undisputedly excluded the malignant diseases.

In order to reduce biases and limitations from the benign etiologies we focused on chronic pancreatitis, postoperative stricture and iatrogenic trauma where every examined modality can have a role in the treatment.

Several publications and randomized studies exist about short term results of the treatment of benign bile duct strictures.[34, 35] These articles shows promising clinical success rates of the endoscopic treatment but the results are still hardly acceptable in a benign disease. As above mentioned, randomized controlled prospective studies focusing on the long term results of the treatment of benign biliary stenosis are not published in the literature.

There are different treatment procedures available for bile duct stenosis but the gold standard method is still not defined. According to the clinical practice endoscopic treatment and stent implantation with or without balloon dilatation is widely used as first line therapy, since it is effective, safe, noninvasive and repeatable. Dilation of strictures is mainly used as a supplementary technique before stent insertion and rarely as a single method. In case of benign strictures plastic stents are the first choice, and non-covered self-expandable metal stents are almost exclusively used in malignant diseases. According to the paper of Katanuma et al. single plastic stent insertion usually does not achieve good short and long term results in terms of benign biliary stricture resolution due to their limited stent diameter, single plastic stents have only short-term patency rates [36]. Multiple stenting published by Costamagna is a more aggressive treatment associated with better results[37] [38]. Although some authors are preferring multiple plastic stent insertion due to the longer occlusion free survival [39], these procedures also have limitations: patient compliance is needed and the risk of stent related complications such as cholangitis is higher. Multiple plastic stents provide bigger lumen size than single stents because the lumen of the implanted stents adds up and the more stent is implanted the less chance is there for an obstruction. There are wide variety of complications that have been reported from the harmless transient stent clogging to severe cholangitis and death.

However, the number of publications about partially or fully covered metal stents used in benign cases are increasing although the indication of these prosthesis are still controversial [3, 14, 40, 41]. Covered self-expandable metal stents appear as a good alternative option, since they have an increased radial diameter, longer stent patency compared to single plastic stents, easier insertion technique and similar efficacy. It should be noted that stent migration is the major problem of fully covered self-expanding metal stents (FCSEMS) [42]. To minimize stent migration, numerous technical modification was done. However, data that clearly demonstrate the superiority of FCSEMS over multiple plastic stenting are lacking[43]. There are also several technical situations where endoscopic procedure cannot be carried out such as a stricture of a bilio-digestive anastomosis which can make endoscopic intervention cumbersome compared to surgery[44].

According to the above mentioned there is a lack of published comparisons and randomized trials, so the acceptable long term effectivity of endoscopic treatment is still doubtful. Due to the heterogeneity of the disease we can get result in a wide spectrum. Cholestasis in patients with chronic pancreatitis may be remedied by endoscopic or surgical means, although endoscopic stent therapy is of lasting success for more than 12 months in only one-third of patients (English language version of the S3-consensus guidelines on chronic pancreatitis: Definition, etiology, diagnostic examinations, medical, endoscopic and surgical management of chronic pancreatitis—Englischsprachige Version der S3-Leitlinie Chronische Pankreatitis A. Hoffmeister1 et al.). A prospective study by Kahl showed an even poorer long-term effect of stent therapy of benign biliary stricture associated to calcifying chronic pancreatitis [29, 45]. The german guideline recommends surgical intervention, if symptoms or cholestasis persist after temporary endoscopic therapy (Level of evidence grade 2b, recommendation grade B, strong consensus)[45].

The role of percutaneous intervention is disputed as well. While endoscopic treatment of patients with previous surgery is technically difficult and anatomical location of the stricture can also result in a low success rate, percutaneous treatment has to be an option in referral centers with adequate case volume and expertise. The use of extracorporeal drain may affect patient's quality of life. PTD should be an option in cases, where surgery is unsuitable due to severe comorbidities or technical challenge such as portal hypertension. The modality allows almost all techniques used in endoscopy: balloon dilatation, stent implantation.

New technologies—such as biodegradable stents or intraductal radiofrequency ablation—are under development to overcome the drawbacks of the existing procedures but there are no evidence about their long term efficacy on human population.[46–48] [49]

Initiated by the previously mentioned difficulties of treatments and lack of clinical data with high evidence we compared several treatment possibilities to find out which is the most effective in long term. Retrospective and prospective studies were also included, both type of studies can give evidences with acceptable and comparable quality in term of long term success rate. Related to that topic some other questions would be interesting as minor and major complications, quality of life etc, but the published data of the selected publications, the retrospective way of data collection does not allow us compare data and frame conclusions in these terms.

Many studies imply that metal or plastic stenting, PTD or surgery is the optimal procedure but none of them reached a consensus and the question remained unanswered. It should be noted that the aim of that study is to compare long term stricture resolution rate of different modalities. However, it is clear that long term disease free survival alone cannot turns the scales. Other parameters such as clinical success rate, complications, life quality, finances influencing the argument should be considered as well. We reviewed a numerous studies written in this topic to find out the answer. As we filtered the articles and compared the results we faced many obstacles that made our results limited. All articles were nonrandomized, the methods applied in the studies were not standard and straight forward and the patient data was often retrospective. Some of them started with one therapy and during the follow up time used other one (if the implanted stent did not function the patient went under PTD or operation), the articles often do not detail the material and the number of the used prosthesis and the exchange frequency do not follow a strict protocol. Because of the retrospective fashion of the articles the follow up times were not standard the patients were followed as long as it was possible and in the publication mean or median follow up time was stated. These complaints show the poor quality of the current literature and all these difficulties made the meta-analysis troublesome.

To be able to make conclusions first we had to exclude articles because of the above mentioned limitations. The widely diversified diseases that lead to the stricture could not be taken into consideration either and the patients had to be managed as one homogenous cohort. Interpreting the long term success rate the unsuccessful primary interventions were not included into the final result in numerous publications typically with endoscopic interventions. Ignoring the initially non-treatable patients the long term success rate is difficult to compare in the different modalities. Due to the lack of available data the statistical comparison is hardly possible. However, adjudication of a success rate of an intervention should be influenced by the number of initially unsuccessful treatments indicating other—possibly successful therapeutic modality. On the other hand, comparison of data analyzing long term success rate according to originally published data and modified long term success rate described previously did not result in a significant difference.

Even within the same study the follow up times varied in a very wide range. The main limitation of this meta-analysis is the comparability of the selected studies. We made the same statistical analysis using an alternative weighting method and a conventional one to be able to make allowance for different follow-up times. Finally, comparing the results of the conventional weighting and the one altered by the follow up time previously described in the methods, the conclusions are robust concerning this difference. The two weighting methods yields almost exactly the same estimates and therefore do not affect the conclusion of the analysis.

However, adjudication of a success rate of an intervention should be influenced by the number of initially unsuccessful treatments indicating other—possibly successful therapeutic modality. On the other hand, comparison of data analyzing long term success rate according to originally published data and modified long term success rate described previously did not result in a significant difference.

In the null hypothesis it was expected that surgery will be as effective as the endoscopic treatment but on the long term it will provide better results. After the meta-analysis of the literature we concluded that all compared methods are superior to the single plastic stent placement. Verifying the opinion of the specialists and the results of numerous non-randomized publications the use of single plastic stent is not recommended. Surgery resulted in the highest long term stricture resolution rate with 84%, followed by multiple plastic stent insertion with 79%, the percutaneous transhepatic treatment and the and covered SEMS with 75%, however the difference was not significant. Thus, the main question is still unanswered, further randomized studies are required. Generally, long term stricture resolution rate irrespectively of the therapy is still not more than 84%. Considering the benign behavior of the disease and the life expectancy of the patients it is still unacceptably low. Therefore, the question should be remain a seething field in the future!

## Conclusion

For further results more investigations are needed but only in consideration of the before mentioned limitations. Endoscopic treatment shows comparable long term patency compared to surgical treatment and seems to have priority in first line treatment due to the lower risk of complications, easy implementation, cost-effectiveness. However, according to the best long term stricture resolution rate appropriate early surgical treatment should be considered for patients with complicated biliary- and anastomotic strictures or chronic pancreatitis as not only second line treatment after endoscopy fails but as initial treatment as well. BBS should be managed by a multidisciplinary team comprising hepatobiliary surgeons, interventional radiologists and endoscopists. Considering the unacceptable long term recurrence rate, in the near future not only new techniques but also other therapies involving new devices are needed in clinical practice.

## Supporting Information

S1 Fig Regression of Logit Success rate on Follow up time (years). (TIF)

S2 Fig Meta regression results of association between logit success rate and follow up time (years) by subgroups. (TIF)

S1 Checklist Prisma 2009 Checklist. (DOCX)
