# Supplementary figures and images for: Meta-Analysis of the Long Term Success Rate of Different Interventions in Benign Biliary Strictures
Source: PLoS One. 2017 Jan 11;12(1):e0169618. doi: 10.1371/journal.pone.0169618 (PMC5226728; doi:10.1371/journal.pone.0169618)

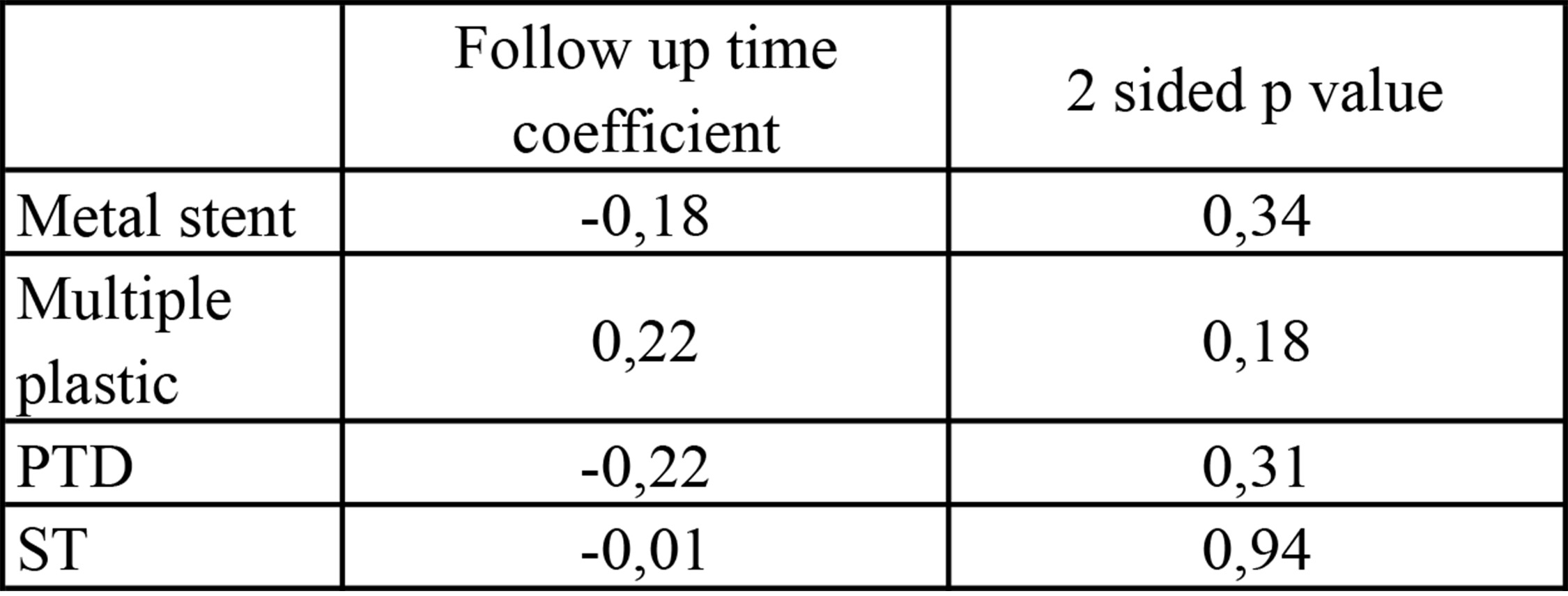

Supplement: S1 Fig — (TIF) [file pone.0169618.s001.tif]

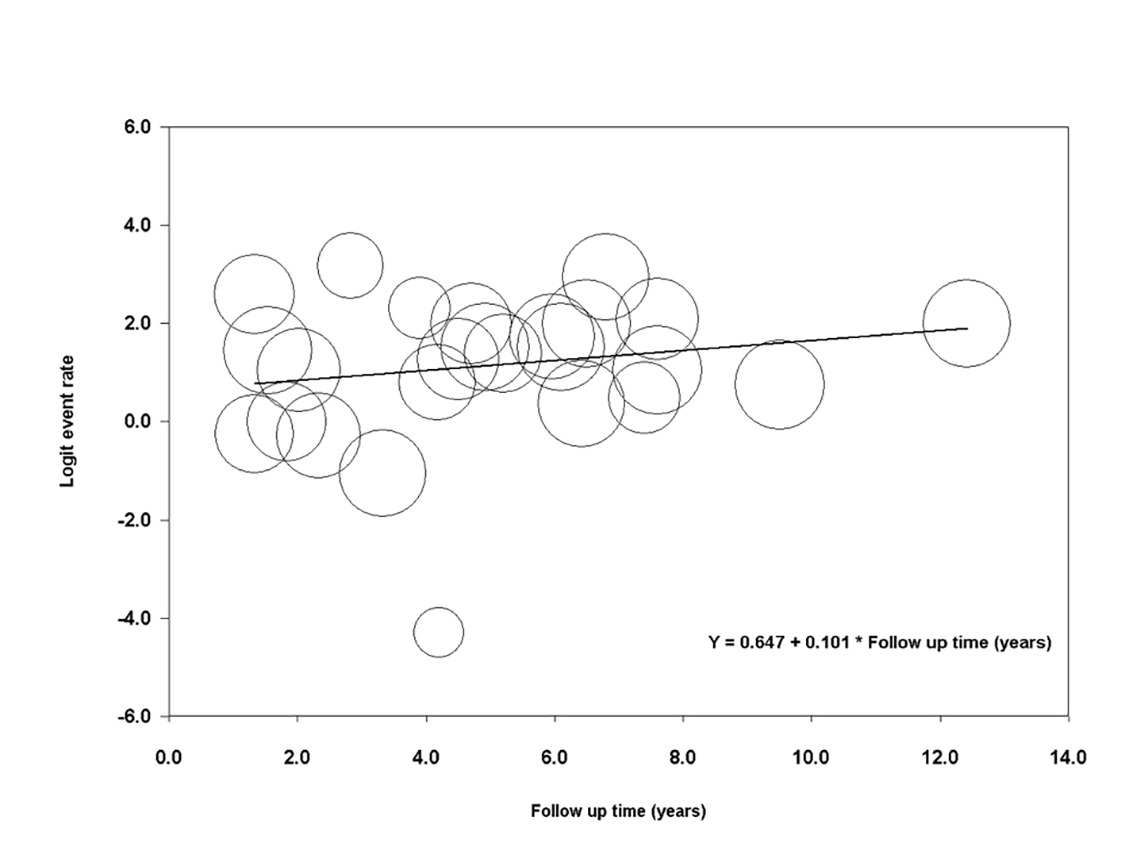

Supplement: S2 Fig — (TIF) [file pone.0169618.s002.tif]
